# So, what's best? Accuracy and acceptance of thermometers in triage and inpatients in a low-resource tropical setting – The MaTe study

**DOI:** 10.1016/j.heliyon.2024.e25806

**Published:** 2024-02-06

**Authors:** Alexandra Turnbull, Harry Putnam, Issa Sesay, Aminata Bangura, Emily Bailey, Jan Henk Dubbink, Martin P. Grobusch

**Affiliations:** a Masanga Medical Research Unit (MMRU) and Masanga Hospital, Sierra Leone; b Center of Tropical Medicine and Travel Medicine, Department of Infectious Diseases, Amsterdam University Medical Centresthe Netherlands; c Centre de Recherches Médicales en Lambaréné (CERMEL), Lambaréné, Gabon; d Institute of Infectious Diseases and Molecular Medicine, University of Cape Town, Cape Town, South Africa; e Institut für Tropenmedizin, University of Tübingen, and German Centre for Infection Research (DZIF), Tübingen, Germany

**Keywords:** Body temperature, Fever, Sierra Leone, Thermometry, Tympanic membrane thermometry, Axillary thermometry, Rectal thermometry, Non-contact infrared thermometry

## Abstract

**Objectives:**

We searched for the most-suitable thermometry method in the low-resource, tropical setting of Sierra Leone, both in terms of accuracy and also patient and user acceptance.

**Methods:**

We conducted a prospective comparative study of different methods of body temperature measurement. Each participant had their temperature taken by four different methods: non-contact infrared temperature (NCIT), axillary, tympanic membrane and rectal measurements. Rectal temperature was considered clinical gold standard. Primary outcome was predicted sensitivity and specificity of thermometry methods in detecting fever (rectal temperature ≥38.0 °C). Questionnaires were used to explore patient and healthcare worker attitudes towards different methods of temperature-taking.

**Results:**

824 rectal body temperature readings were taken from 562 participants. The mean rectal temperature was 37.4 °C (IQR 37 °C to 37.7 °C), with a minimum reading of 35.2 °C and maximum of 41.0 °C. Tympanic membrane thermometry showed the highest sensitivity of fever detection using the Genius3 TM thermometer (sensitivity 70.8 %, 95 % CI 60.2%–79.9 %; specificity 97.2 %, 95 % CI 95.5–98.4 %); and Braun TM (sensitivity 51.5 %, 95 % CI 42.6%–62.0 %; specificity 98.8 %, 95 % CI 97.7–99.5). NCIT thermometry sensitivity was low (36.8 %–41.4 % for the two devices used). Axillary thermometry sensitivity was 40.6 %. Participants ranked NCIT as the most and rectal as the least preferred method. Questionnaires from 32 participating nurses showed agreeability to using NCIT, TM and axillary methods routinely, but less so for rectal thermometry.

**Conclusions:**

When combining the accuracy of different thermometry methods in detecting fever with user and patient acceptability, tympanic membrane thermometry appears most suitable but also has limitations.

## 1 Introduction

Despite some progress, communicable diseases remain amongst the top causes of death worldwide [1]. In the African region, over 40 % of deaths in 2016 were due to infectious diseases. Lower respiratory tract infections, diarrhoea, HIV, tuberculosis and malaria remain amongst the leading causes of mortality in the region [2]. Furthermore, especially in the West Africa region, viral haemorrhagic fevers (VHF) including Ebola, Marburg and Lassa fever remain constant and serious public health threats.

In low-resource settings with limited diagnostic capabilities, early and accurate recognition of fever is essential for the diagnosis of these diseases, for the prevention of hospital acquired infection and for the protection of healthcare workers. The COVID-19 pandemic has further highlighted the need for effective triage and screening systems, with fever recognition being central to this.

Fever is a pyrogen-mediated rise in body temperature above normal, considered as a thermoregulatory manifestation of the immune response [3]. However, this simple definition does not capture the pathophysiological complexity of what might be a deviation from ‘normal body temperature’, which itself is subject to variation with regard to sex, diurnal variation, and whose readings differ depending on the site where it is measured, and with which device. ‘Normal’ core body temperature varies with time of day, level of activity and environmental conditions [4,5] and is subject to hormonal influences; let alone that there is not one single body temperature at a time across the whole organism [3]. As such, there is also no universally accepted numerical definition of fever. The most-widely accepted threshold irrespective of the method of measurement in clinical practice appears to be ≥ 38.0 °C across countries of differing socio-economic status and differing climates. However, the measurement of body temperature itself (with the methods which theoretically exist not necessarily equalling those practically available in all settings) is an ongoing subject of debate. Sajadi and Romanowsky [3] provide a comprehensive overview on available techniques. Without doubt, measurement of core body temperature via pulmonary artery temperature taking remains the ‘true’ gold standard, which is nowhere in the world feasible outside of intensive care settings.

In most Sierra Leonean hospitals, current practice is to use non-contact infrared thermometers (NCITs) for all temperature measurements on babies, children and adults. The use of NCITs is increasingly common in hospitals and triage settings, especially during the COVID-19 pandemic. There are many presumed benefits of NCITs, including reduced risk of patient-to-patient and patient-to-staff transmission of infectious agents, ease and speed of use, reduced reader bias through digital display and their non-invasive nature [6]. Other anatomical sites for body temperature measurement used commonly in healthcare settings include rectum (thought as of gold standard for routine clinical practice), axilla, oral cavity and the tympanic membrane.

Despite these potential benefits of NCITs, thermometry methods for use in triage and inpatient wards need to be accurate. Evidence on this is lacking for many geographical regions, including West Africa. A recent and extensive systematic review and meta-analysis on the accuracy of NCITs for the detection of fever found relatively good pooled sensitivity and specificity of 0.781 (95 % CI 0.628–0.882) and 0.926 (95 % CI 0.799–0.975), respectively, but acknowledges that environmental factors including ambient temperature and humidity can significantly affect accuracy [7]. Of 30 studies found and analysed, 19 were included for analysis. Of note, none of the studies included were from Africa or from any low-income, tropical setting. Those included from hot climates were almost exclusively in air-conditioned facilities. Similar studies have assessed accuracy of thermometry methods in similar climates [8,9] but did not include NCIT. A recent study on 200 outpatient participants attending a secondary care hospital in Nigeria showed low sensitivity (13 %) of NCITs to detect fever ≥38.0 °C, however, used oral electronic thermometry as the reference method [10].

Choice of method of regular thermometry also needs to be acceptable to the patient and care provider. Evidence here is also lacking in the West African region. One Nigerian study examined mothers’ preferences between rectal, axilla and temporal artery temperature measurement for their children. No statistically significant preference was identified [11]. No identified studies have examined adult or nursing staff opinion and none have compared NCIT to more invasive methods.

We hypothesise that NCITs that are commonly use in Sierra Leone and West Africa are unreliable for the detection of fever. We expect tympanic or axillary methods to be more accurate and equally acceptable to patient and staff. Despite being gold standard, we predict rectal thermometry is not an accepted long-term method of thermometry.

With this study, we propose to establish the most suitable method of measure body temperature in low-resource, tropical settings by comparing different common methods of temperature measurement.

## 2 Methods

### 2.1 Setting

This prospective comparative study was run at Masanga Hospital, a 120-bed general hospital in Tonkolili District of Northern Sierra Leone over a period of four weeks in May/June 2022. It was ran at the end of the Sierra Leone dry season when conditions are amongst their harshest, with ambient temperatures above 30 °C and relative humidity around 80 % on average [12].

Patients attending Masanga triage facility were studied in the mornings (08:15 to 12:30) and inpatients on the acute adult, paediatric, and obstetric wards were studied in the afternoons (14:00–16:00). These times were chosen to maximise participant enrolment and fever recording because of high volumes of patients attending triage in the morning and incidence of fever is known to be greater later in the day [13].

The study had the specific objective to try and reflect ‘real-life’ hospital practice in Sierra Leone and West Africa – measurements were taken in genuine hospital triage facilities and the same type of thermometer was used for babies, children and adults. In line with a combination of NICE guidelines and the American Academy of Paediatrics, children under the age of six months were excluded, as the use of tympanic membrane thermometry is not recommended in this age group [14,15].

### 2.2 Ethics approval and participant consent

Ethical approval was granted by the Sierra Leone National Ethics and Scientific Review Committee on 11 May 2021 (no document number issued). The LSHTM Ethics Committee also granted ethical approval (LSHTM MSc Ethics Ref: 25680) and their Research Governance and Integrity Office kindly provided sponsorship. All participants or their legal guardians provided informed consent to participate in the study (also see full ethics statement at end of manuscript).

All potentially eligible babies, children and adults were approached following the routine triage process at Masanga Hospital by our study team research assistant. Adults or parents of eligible children and babies were consented through information sheets, and the study was explained to them with the aid of picture cards (see Supplementary Fig. 1). Written consent by signature or thumbprint was obtained by all participants or by their guardians. An assent form was used for those aged between sixteen and eighteen, requiring both teenager and adult consent. Due care and attention were paid to the sensitivity of rectal temperature measurements with further consent having been sought at the time of carrying out the procedure. Temperature taking was stopped if the participant showed hesitancy or unexpected discomfort.

### 2.3 Data collection

Following consent, individuals were brought into a private room next to the triage facility. Consecutive temperature recordings were taken in quick succession with all participants being subjected to all six different measurement devices. Rectal temperature was used as the reference method. Temperature measurements were taken in a particular order, from least to most invasive, as follows: Infrared Digital Forehead Thermometer ‘HTD8808’ (P&C Electronic Technology Co, Ltd, Shenzhen, China), Infrared Non-Contact Thermometer HT706 (MIEO, Huafeng Shenzhen, China), iProven Digital Thermometer DTR-1221A (USA) used in the axilla, Genius™ 3 Tympanic Thermometer (COVIDEAN, China), Braun Thermoscan® IRT3030 Infrared Ear Thermometer (Kaz Europe Sarl, Lausanne, Switzerland), iProven Digital Thermometer DTR-1221A (USA) used rectally. See Supplementary Fig. 2 for full questionnaire and data documentation.

Inpatients had their temperatures taken daily on the wards in the afternoon via the same methods in the same order. Privacy measures were put in place. Verbal consent was re-taken daily before each repeat reading. The opinion questionnaire was carried out only after the first set of temperature measurements for inpatients.

All consenting, available nursing staff were enrolled. Thermometry methods were explained, after which they carried out each method on a consenting study participant. Following this they were asked to complete a questionnaire to assess nursing staff views to different methods (see Supplementary Fig. 3).

All thermometers were set up and used as per product specific instructions. A mixture of imported and locally sourced thermometers were used to help maximise and enable implementation of our research findings with all participants being subjected to all six devices. In Sierra Leone, TM thermometers are rarely used and very difficult to source even within the capital city; therefore, both TM thermometers were imported – Genius3 is seen to be a ‘high-end’ TM thermometer with a retail price of £230/€260. The Braun TM sells at £30/€34. NCIT's are widely found in Sierra Leone, with the MIEO being sold in several local pharmacies costing approximately £15/€17, HTD 8808 costing £32/€36, and the iProven Digital Thermometer DTR-1221A costing £8.50/€10 (all prices as per time of writing end of November 2023).

### 2.4 Data storage and handling

All data was entered onto paper records. Anonymisation was ensured by all personal data, including temperature measurements, being affiliated with a study number, rather than a name.

EpiData (EpiData Software, 2021; http://www.epidata.dk) database was used, and all results were finalised using double data entry and StataSE 17 was used for data analysis. Fever was defined as ≥38.0 °C rectally. For the axillary thermometer, however, we have used fever thresholds of both ≥38.0 °C and ≥37.5 °C (maintaining a rectal fever definition of ≥38.0 °C) given evidence put forward by a WHO expert study group who recommend an axillary fever threshold of 37.5 °C for best accuracy in detecting fever [9,16].

## 3 Results

### 3.1 Basic demographic data

In total, 562 participants were enrolled into the study - some of which had several sets of temperature recordings taken on consecutive days, totalling 824 rectal (gold standard) measurements. The mean rectal temperature was 37.4 °C (IQR 37 °C–37.7 °C), with a minimum reading of 35.2 °C and maximum of 41.0 °C. Of 824 readings, 133 recorded a fever of ≥38 °C. A full summary of participant demographic data is shown in Table 1.

**Table 1:** Demographic data.

Variable	Summary
**Number of participants, n**	562
Adults, n (%)	413 (73.5)
Children, n (%)	149 (26.5)
**Median age (years), (IQR)**	25 (12–40)
Adults, n	31, (23–45)
Children, n	2, (1–4)
**Sex (female), n (%)**	361 (64.2)
Children, n (%)	75 (50.3)
Adults, n (%)	286 (69.2)
Pregnant (adult/female), n (%)	125 (43.7)
**Total rectal readings, n**	824
**Rectal fever ≥38.0**^**0**^**C, n (%)**	133 (16.1)
Adult, n (%)	46 (9.0)
Children, n (%)	91 (28.9)
**Location, n**	824
Triage, n (%)	446 (54.1)
Ward, n (%)	378 (45.9)
**Black African ethnicity, n (%)**	824 (100)

Due to ‘error’ readings, primarily with the Genius3 TM thermometer (which is programmed to stop functioning at high ambient temperatures), not all sets of temperature recordings were complete. Rectal, axillary and both NCITs had all 824 readings. The Genius3 TM thermometer had a total of 656 readings and Braun TM thermometer 815 with error messages being displayed exclusively in the afternoon recordings when ambient temperature was highest.

### 3.2 Detection of fever

The primary objective of the study was to calculate the accuracy of various thermometers in detecting fever. The thermometer with the highest sensitivity was the Genius3 TM thermometer, with a sensitivity and specificity of 70.8 % (95 % CI 60.2 %–79.9 %) and 97.2 % (95 % CI 95.5 %–98.4 %), respectively. The other TM thermometer (Braun) had a sensitivity of 51.5 % and specificity of 98.8 %. The axillary and both NCITs had relatively low sensitivities of 40.6 %, 41.4 % (HTD) and 36.8 % (MIEO). Table 2 shows full sensitivities, specificities, positive predictive values (PPV) and negative predictive values (NPV) for all thermometer devices.

**Table 2:** Sensitivity, specificity, PPV, NPV of all thermometer devices in the detection of fever defined as ≥38 °C.

	Genius3	Braun IRT3030	iProven	HTD 8808	HT706 MIEO
	TM	TM	Axilla	NCIT	NCIT
**Sensitivity, %**	70.8	51.5	40.6	41.4	36.8
(95 % CI)	(60.2–79.9)	(42.6–60.0)	(32.2–49.5)	(32.9–50.2)	(28.6–46.0)
**Specificity, %**	97.2	98.8	99.9	95.7	98.1
(95 % CI)	(95.5–98.4)	(97.7–99.5)	(99.2–99.9)	(93.9–97.1)	(97.2–99.2)
PPV, %	79.8	89.3	99.2	64.7	81.7
(95 % CI)	(69.2–88.0)	(80.0–95.3)	(90.3–99.9)	(53.6–74.8)	(69.6–90.5)
NPV, %	95.5	91.5	89.7	89.5	89.0
(95 %CI)	(93.5–97.0)	(89.3–93.4)	(87.4–91.8)	(87.0–91.6)	(86.6–91.1)

With a fever definition of ≥37.5 °C for axillary thermometry (when compared to rectal thermometry with a fever definition of 38.0 °C), the axillary thermometer showed a much higher sensitivity of 63.9 % (95 % CI 55.1%–72.0 %) vs 40.6 % (95 % CI 32.2 %–49.5 %) for a fever threshold of ≥38.0 °C. For the lower ≥37.5 °C fever threshold, specificity was 97.8 % (95 % CI 96.5%–97.8 %), PPV was 85.0 % (95 % CI 76.5%–91.4 %) and NPV 93.4 % (95 % CI 91.3%–95.1 %). In comparison, with a fever threshold of ≥38.0 °C for the axillary thermometer, specificity was 99.9 % (95 % CI 92%–99 %), PPV was 99.2 % (95 % CI 90.3%–99.9 %) and NPV was 89.7 % (95 % CI 87.4%–91.8 %).

The temperature recorded during the routine triage process at Masanga Hospital by the triage nurse, just prior to study enrolment and temperature-taking, was also recorded. This was to consider the performance of NCITs when used in a less controlled manner outside a study site context. Triage fever detection sensitivity for the NCIT used at Masanga Hospital was 19.56 % (95 % CI 9.4 %–33.9 %), and specificity was 99.7 % (95 % CI 98.6%–99.9 %).

The sensitivity, specificity, PPV and NPV were also calculated when separating adults from children (Table 3). The 95 % confidence intervals tended to be larger, given the smaller study sample and lower number of fevers per group. The largest difference in sensitivity between adults and children was the Genius3 TM thermometer, which had a sensitivity of 52.9 % (95 % CI of 27.8%–77 %) for children versus 75 % (95 % CI 63.4–84.5) for adults. The Braun TM thermometer had similar sensitivities on adults and children, with 50.5 % and 53.8 %, respectively. The PPV was markedly reduced in the Genius3 TM thermometer in children at 52.9 %, and the HTD axillary readings also produced a low PPV of 50 %. Sensitivities for the NCIT thermometers remained low with a sensitivity of 33.3 % (95 % CI 19.6%–49.5 %) for children with the MIEO and 38.1 % (95 % CI 32.5%–53.7 % for the HTD.

**Table 3:** Sensitivity, specificity, PPV and NPV of all thermometer devices in the detection of fever defined as ≥38 °C, separated into adults versus children.

	Genius3	Genius3	BraunIRT3030	BraunIRT3030	iProven	iProven	HTD8808	HTD8808	MIEO	MIEO
	TM	TM	TM	TM	Axilla	Axilla	NICT	NCIT	NCIT	NCIT
	Adult	Child	Adult	Child	Adult	Child	Adult	Child	Adult	Child
**Sensitivity %**	75	52.9	50.5	53.8	42.9	35.7	42.8	38.1	38.5	33.3
(95 % CI)	(63.4–84.5)	(27.8–77.0)	(39.8–61.2)	(37.1–69.9)	(32.5–53.6)	(21.6–52.0)	(32.5–53.7)	(32.5–53.7)	(28.4–49.2)	(19.6–49.5)
**Specificity %**	95.4	98.0	97.8	99.4	100	99.8	93.8	96.6	97.8	98.7
(95 % CI)	(91.1–98.0)	(96.0–99.1)	(94.9–99.3)	(81.1–98.1)	(98.4–100)	(98.8–99.9)	(89.7–96.5)	(94.5–98.0)	(94.9–99.3)	(97.2–99.5)
PPV, %	87.0	52.9	90.2	87.5	100	93.8	73.6	50.0	87.5	70
(95 % CI)	(75.1–94.3)	(27.8–77.0)	(78.6–96.7)	(67.6–97.3)	(91.0–100)	(70.0–99.8)	(59.7–84.7)	(31.9–68.1)	(73.2–95.8)	(45.7–88.1)
NPV, %	90.2	98.0	83.0	96.3	81.1	94.5	80.2	94.5	79.6	94.3
(95 %CI)	(84.9–94.1)	(96.0–99.1)	(77.9–87.3)	(94.1–97.7)	(76.0–85.6)	(92.1–96.4)	(74.8–84.8)	(92.3–96.2)	(74.4–84.2)	(91.8–96.2)

### 3.3 Mean differences

Mean differences between rectal thermometry and other thermometry methods tended to show that rectal thermometry reads higher than the comparator - with one exception being the HTD thermometer, which had a mean difference of −0.20 °C. Axillary measurement had the largest mean difference of devices tested at 0.80 °C. Table 4 shows a summarised version of mean differences, with confidence intervals and p-values of each device compared to rectal temperature. All mean differences were statistically significant with p-values <0.001.

**Table 4:** Mean, mean difference (to rectal temperature) and p-value of thermometry devices (paired T-test).

	Rectal	Genius3	Braun IRT 3030	iProven	HTD 8808	MIEO
		TM	TM	Axilla	NCIT	NCIT
**Mean,**^**0**^**C**	37.4	37.0	37.0	36.6	37.6	37.0
(SD), ^0^C	0.73	0.84	0.74	0.80	0.74	0.67
**Mean difference,**^**0**^**C**		0.32	0.40	0.80	−0.20	0.43
(95 % CI), ^0^C		(0.27–0.37)	(0.36–0.44)	(0.75–0.83)	(-0.14 to −0.25)	(0.38–0.47)
**P-value**		<0.001	<000.1	<0.001	<0.001	<0.001

**Fig. 1:**
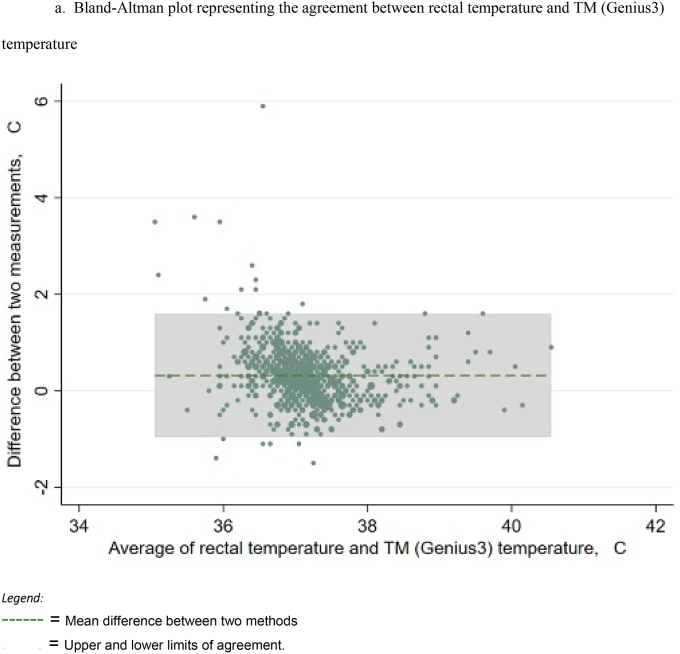

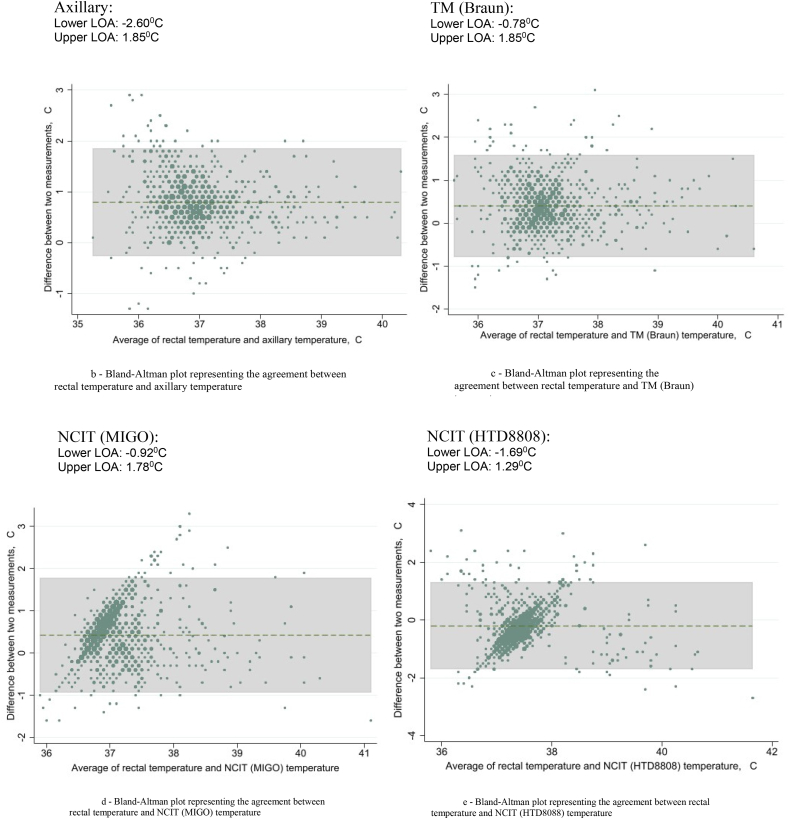
Bland-Altman plots representing agreements between rectal temperature and various thermometry methods.

For further analysis of the agreement between rectal thermometry and the other thermometer devices, Bland-Altman analysis was performed. Fig. 1a shows the Bland-Altman plot for agreement between the rectal and Genius3 TM thermometers, showing a mean difference of 0.32 °C with the upper and lower limits of agreement shaded in grey. Limits of agreement were calculated (mean difference ± 1.96 SD) and all plots with upper & lower limits of agreement are displayed in Fig. 1b–e. Interpretation of this means that, in 95 % of cases, you can expect the difference between the rectal temperature and Genius3 temperature to range between 0.96 °C lower and 1.60 °C higher than the average of both measurements of temperature.

Having calculated the average mean difference of each thermometer device to ‘gold standard’ rectal temperature, we measured the effect of adjusting all measurements taken by the mean difference and from that, calculated further sensitivities, specificities, NPV and PPV (Table 5). When accounting for mean difference, sensitivity and NPV increased in all but the HTD 8808 NCIT but had notable negative effects on both the specificity and PPV.

**Table 5:** Mean adjusted sensitivity, specificity, PPV and NPV results. of all thermometer devices in the detection of fever defined as ≥38^*0*^C. Original results displayed below for easy comparison.

	Genius3	Braun IRT3030	iProven	HTD 8808	HT706 MIEO
	TM	TM	Axilla	NCIT	NCIT
**Mean adjusted sensitivity, %**	78	68	80	34	46
Original sensitivity, %	*70.8*	*51.5*	*40.6*	*41.4*	*36.8*
**Mean adjusted specificity, %**	91	93	93	99	92
Original specificity, %	*97.2*	*98.9*	*99.9*	*95.7*	*98.1*
**Mean adjusted PPV, %**	58	65	70	88	53
Original PPV, %	*79.8*	*89.3*	*99.2*	*64.7*	*81.7*
**Mean adjusted NPV, %**	96	94	96	89	90
Original NPV, %	*95.5*	*91.5*	89.7	*89.5*	*89.0*

### 3.4 Patient preferencing

**Fig. 2:**
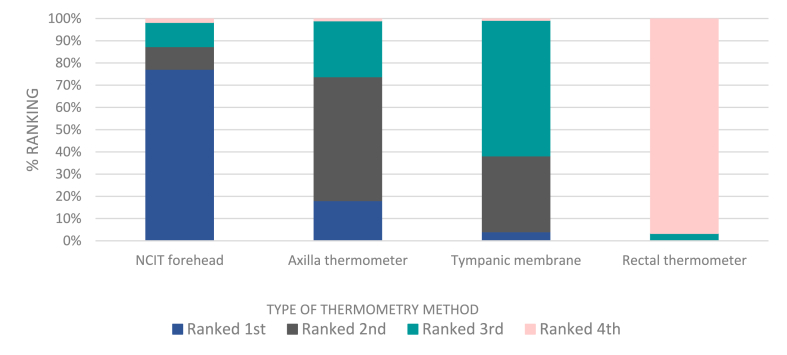
Participant preference of each thermometer device, % rankings.

Following temperature measurements, participants were asked to rank (one to four) the four types of thermometry by preference, with ‘1’ being most preferred and ‘4’ least preferred. All adults ranked their preferences with most participants preferring the NCIT, 319 ranked it first and only seven participants last - creating a mean rank of 1.37. Rectal thermometry was notably least preferred (399 ranking it last), with a mean rank of 3.96. The axillary method had a mean rank of 2.09 and the TM had a mean rank of 2.59. Fig. 2 shows the ranking percentages of each method of thermometry. Friedman Test showed these differences to be statistically significant (p < 0.001).

Participants were also asked whether they would be comfortable with each thermometry method to be used regularly (up to three times per day) on themselves, as if they were an inpatient on a ward. Participants largely responded ‘Yes’ to this question for axillary, NCIT and TM methods. Despite being least preferred, half (49.9 %) of participants also responded ‘Yes’ to feeling comfortable with regular rectal measurements, but notably less so than the other three methods. Fig. 4 summarises these findings, with all preferences being statistically different from one another with the exception of axillary to NICT.

If a participant was aged less than 16, then one parent/guardian was asked similar questions (to preference rank and their feelings towards regular use of each type of thermometer) but relating to use on their child, rather than themselves. All 149 parents/guardians (100 %) of child participants were happy for axillary, tympanic membrane and NCITs to be used on their children regularly. 135 of the 149 parents (90.6 %) reported they would be comfortable for rectal thermometry to be used regularly on the wards and at triage; this was much higher than the responses from the adults. Ranking in children was similar to ranking in adults, with parents ranking NCIT method their favourite (mean rank of 1.3) and rectal ranking least favourite with a mean rank of 3.9. Mean ranking for axillary and TM thermometry was 2.1 and 2.6, respectively.

### 3.5 Nurses’ preferencing

Thirty-two nursing staff from both adult and paediatric wards at Masanga Hospital were enrolled and asked questions regarding their preferences to thermometer use. To account for the possibility that not all nursing staff would be familiar with all four types of thermometry methods, nurses were shown, guided and watched as they used all four methods on a consenting participant prior to answering the questions. Nurses were also asked whether they would be happy to use each type of thermometer as their regular thermometry method whilst taking their routine observations on adults and children separately.

**Fig. 3:**
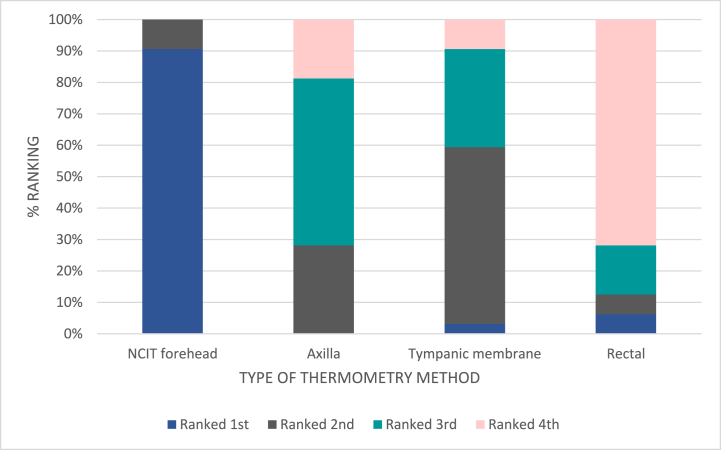
Nursing preference of each thermometer device, % rankings.

Of 32 enrolled nurses, 29 ranked the NCIT first and 23 of 32 nurses ranked the rectal thermometer last. No nurses ranked NCIT as their third or least favourite method. Most nurses ranked axillary and TM thermometry in the middle, with slight preference between the two leaning towards the tympanic membrane method. This can be seen in Fig. 3, with 56 % of nurses ranking TM second and 31 % of nurses ranking it third versus 27 % of nurses ranking axillary second and 53 % ranking it third. Statistical analysis showed Friedman p < 0.005 evidencing a statistical significance between the differences.

**Fig. 4:**
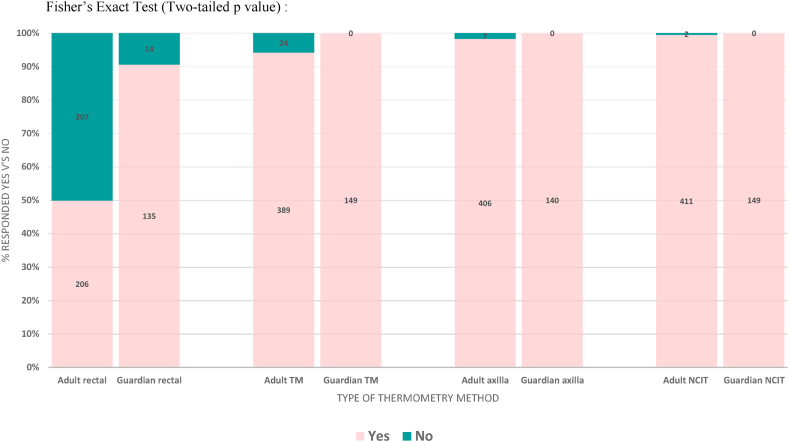
Summary of participants' responses to the question ‘Would you be comfortable to have your temperature taken via these methods regularly, as if you were an inpatient on a ward’.

**Fig. 5:**
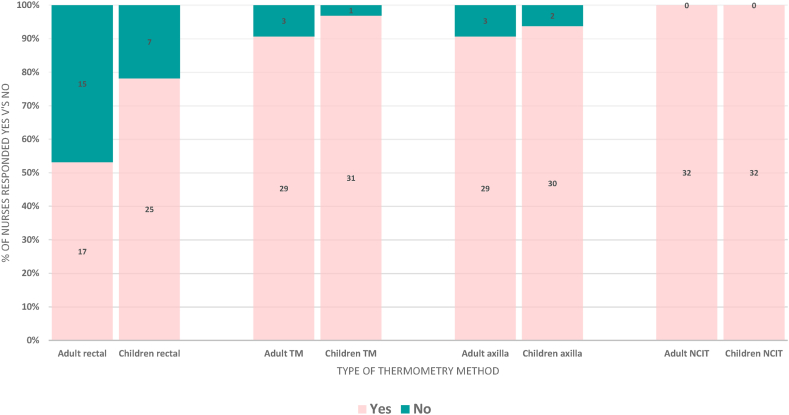
Summary of nursing staff response to whether they would be willing to take patient temperatures via these methods regular (i.e., for routine observations on wards).

Nurses were also asked whether they would be happy to take routine ward temperature observations using the four different methods of thermometry on children and adults. As shown in Fig. 5, for both children and adults, 100 % of nurses were in agreement to use NCITs, and most nurses willing to use the TM and axillary methods, too. 78 % of nurses were confident to regularly use rectal thermometry on children and 53 % being happy to do this on adults.

## 4 Discussion

This prospective comparative study is the first of its kind in the West African setting. Its participants were ward inpatients or attending outpatients at the study hospital. The methodology aimed to recreate, as closely as possible, current and realistic practices within the hospital and therefore account for environmental factors.

We hypothesised that NCITs currently used in common practice would be found to be unreliable for fever detection. Analysis of the results demonstrates this hypothesis to be accurate. When divided into adults' and children’ sub-categories, sensitivities still remained low throughout with the lowest sensitivity being 33 % for the MIEO device in children, suggesting two of every three fever episodes are missed.

Axillary fever detection proved to be similarly poor, showing a sensitivity of 40.6 % only with the largest mean difference to rectal temperature of all devices (0.8 °C), and with the largest lower and upper limits of agreement (−2.60 °C–1.85 °C). However, when applying an axillary fever definition of ≥37.5 °C (as some literature suggests), sensitivity was much improved at 63.9 %, rendering it one of the more sensitive thermometers tested.

Tympanic thermometry was relatively accurate for fever detection, with sensitivities of 70.8 % and 51.5 % for the Genius3 and Braun devices, respectively. According to the Genius3 TM thermometer's product operating manual [17], it has an ambient temperature operating range between 16 °C to 33 °C; out of this ambient temperature range, the thermometer does not perform a temperature reading. However, the sensitivity of the Genius3 does not account for the 168 'error' readings given due to ambient temperature above its functioning threshold. If these error messages were to be considered ‘missed fevers’, the sensitivity would be 47.4 % and such a device would not be able to be used given its ability to stop functioning when operating outside the recommended room temperature. By definition, as the gold standard for comparison, rectal thermometry had a 100 % sensitivity for fever detection.

With regards to acceptability, participant preference was for NCITs, followed by axillary then tympanic measurement. The majority of participants would be happy for these three methods to be used regularly. Rectal thermometry was ranked last in preference. Amongst adults, less than half would consider rectal thermometry to be used on them regularly as acceptable. When posed to parents/guardians of children, however, over 90 % accepted rectal thermometry to be used regularly on their children. At least 90 % of nurses found axillary, tympanic and NCIT methods acceptable for regular use for both adults and children. 78 % found the regular use of rectal temperature measurements acceptable for children, but only 53 % for adults. Contrary to the hypothesis, this therefore demonstrates that rectal thermometry could be considered an accurate and acceptable method of fever detection in children in inpatient and triage settings. Of course, other local considerations, such as privacy, time efficiency and infection control precautions need to be taken into account when formulating guidance on methodology, however.

As expected amongst adults; although gold standard, rectal thermometry is not well accepted by patients or staff. Given this, and using the data we have used, it appears that tympanic thermometry is the best alternative but comes with practical considerations that will be further discussed below.

In a recent meta-analysis, the sensitivities of ten different NCIT devices used was analysed, with a total of 5562 measurements from thirteen different studies. The pooled sensitivity for these devices was 80.8 %, with specificity of 92.0 % [7]. This is roughly double the sensitivity that our study found for either of the NCIT devices used. It should be noted that there was significant heterogeneity in the individual studies included in the meta-analysis. Studies varied in terms of their setting and population, the method and reference range for fever confirmation and the particular NCIT device used. Sensitivities reported by the individual studies analysed ranged from approximately 18 % – 97 %. Given this wide range of reported sensitivities, it is important to consider the potential factors affecting this. Aggarwal et al. acknowledge the potentially significant role that environmental factors, including ambient temperature and relative humidity, may have on the accuracy of these devices, as well as patient factors such as recent exertion or heavy perspiration [7]. With this in mind, our study is an important addition to the data set given it is the first study to assess the accuracy of NCIT in rural West Africa in a setting that mimics the reality of triage and ward environments in the area (*i.e.*, often more rudimentary, exposed settings with no temperature regulation).

In the study presented here, the sensitivities of the two NCIT devices were closely matched. However, there was substantial variation in the mean differences between the devices compared with rectal temperature. Between the two devices, used almost simultaneously on the same participant in the same environment, there was a mean difference of 0.63 °C (95 % CI 0.60 to 0.66, p=<0.05). This demonstrates significant inter-device variability. It should be acknowledged that, in accordance with the manufacturers' guidance, the HTD8808 device was calibrated to rectal temperature prior to commencing the study. This resulted in a +0.7 °C alteration to the device's factory settings which may account for the discrepancy between the two devices. The MIEO device had no similar manufacture recommendations.

We also measured further sensitivities, specificities, NPV and PPV following adjustment of all temperatures with the calculated mean difference; Table 5). When medical facilities are deciding about which thermometry method may be most suitable, they could consider adjusting each temperature reading with the mean difference (or alternatively lowering their fever threshold) should sensitivity and NPV felt to be more important in temperature taking (not appropriate in the HTD 8808 NCIT). Adjusting for mean difference would reduce missed fevers in the triage setting, but it notably reduced the PPV significantly, and to some extent the specificity, too; and hence may be not felt appropriate an appropriate action to take.

We consider the environmental conditions of the current study to be the most likely cause to the low sensitivities of NCITs found in comparison to previous studies. However, given the acknowledged inter-device variability, we cannot rule out quality of equipment as a contributing factor. These devices were chosen for the study because they are the readily available devices in the setting and importing alternatives is logistically challenging, as it is for many similar settings.

Furthermore, user variability may play a significant role in the accuracy of NCIT measurements. In the triage arm of this study, temperature measurements were taken immediately after the participant had been triaged as per standard hospital policy at that time. Triage was performed by one of three regular triage nurses and included temperature measurement using a standard hospital NICT device. Fever detection at triage (defined at NCIT ≥38 °C) had a sensitivity of 19.6 % (95 % CI 9.4 %–33.9 %). This means that current triage methods miss four out of five fevers entering the hospital grounds. Given all other conditions were the same, the discrepancy between triage and research team findings is likely due to user and/or device variation. Owing to their sensitivities of less than 50 % when using best reasonably achievable practices, NCIT is an inappropriate choice of device for fever recognition in triage and inpatients in rural, tropical settings such as this one and in many other parts of the worlds without air conditioning.

Our results on the accuracy of tympanic membrane thermometry for fever detection are comparable with previous literature. In adults, sensitivity of tympanic membrane thermometry at detecting rectal fever ≥38.0 °C have been reported as 74.1 % and 68.3 % in two separate studies based out of New York [18,19]. In the more comparable Nigerian setting, tympanic thermometry was found to have a sensitivity of 76 % and 91.5 % amongst neonates and under-fives, respectively [20,21]. We found TM thermometry to be the most reliable indicator of fever of the peripheral methods trialled, with the Genius3 device having a similar sensitivity to these previous findings. However, when taking into account that the Genius3 device failed to produce readings on 168 occasions owing to excessively high ambient temperatures, a higher specification TM thermometer maybe a suitable device for a tropical setting.

Axillary temperature proved highly specific, with a good positive predictive value but lacked the sensitivity of an effective fever screening tool when using a fever definition of ≥38.0 °C. Results were similar to those found by Abdulkadir et al. in the under-five population in Nigeria [20]. Sensitivity of the axilla thermometer was 63.9 % when using a fever definition of ≥37.5 °C, thus making it similar to the sensitivity of the TM devices. Loss of specificity was minimal (from 99.9 % to 97.8 %). Healthcare settings could consider using axilla thermometers (affordable, easy to access and acceptable to patients and staff) for temperature-taking; however, education would have to be provided given the importance of the altered fever threshold definition.

With regards to user and participant acceptability of different thermometer types, there is little research conducted. As we hypothesised, rectal temperature was not well tolerated other than when solely applied to children. This matches the 2014 study by Kelechi et al. in Nigeria, who found mothers did not have any significant preference in thermometry methods between rectal, axilla and temporal artery measurement [11]. A cohort of parents in Newcastle-upon-Tyne in England (a very different study population to ours) showed quite different views, with 37 of 42 parents showing concern at rectal thermometry [22]. It is re-assuring that TM thermometry, which showed the highest sensitivities of fever detection, was well tolerated by both participants and users, despite this being a new type of thermometry method to most nurses and participants. This is similar to results by Staaij et al. who found TM thermometers to be easy to use (primarily by parents) and well tolerated [23].

To the best of our knowledge, this is one of the first studies of its kind to assess a range of thermometers in a tropical and low-resource setting using rectal thermometry as reference method in terms of the clinical gold standard in this setting. We used local available as well as imported thermometers. The study was designed to mimic realistic clinical practice to produce relevant and applicable results to healthcare facilities in the region. It was a powered study with a large sample size of 824 rectal readings. It combines objective data on thermometer accuracy with subjective data on comfort and cultural acceptability.

## 5 Strengths

This is one of very few studies of its kind to assess NCIT's, TM thermometers and thermometers used in the axilla compared to ‘gold standard’ rectal thermometry in a tropical and under-resourced setting. This study also evaluates both patient and nursing perspectives of all four different thermometry methods which has been rarely evaluated in a setting such as ours. The study was tailored towards clinical practice as much as possible to enable healthcare facilities in Sierra Leone and West Africa to utilise our results and implement our recommendations as much as possible. It was a powered study with a relatively large sample size of 824 rectal readings.

## 6 Limitations

Limitations to this study include the use of rectal temperature as gold standard. Whilst rectal thermometry is widely recognised as the gold standard in routine clinical practice, measurement of core body temperature via pulmonary artery temperature taking remains true gold standard. However, this is not realistically achievable in most clinical settings. We recognise that a range of devices are available for each thermometry method, not all of which were possible to include in this study. Our results therefore generalise the accuracy of all NCITs and TM thermometers based on the readings produced by the models that we used. When pooling our data analysis for adults and children, we had large study numbers and relatively narrow confidence intervals, when these age categories were separate (particularly in children), we had fewer fevers and participants; hence reducing the power and increasing the confidence intervals in our results.

We choose not to use a fixed measurement for distance of the NCIT to the forehead. Study personnel instead estimated the distance in line with manufacturer recommendation. This was felt to be more in line with the reality of routine practice but could be viewed as a limitation by introducing inter-user variability.

Our study was carried out at the end of the dry season in Sierra Leone when humidity levels are very high. It is unknown how much the changing levels of humidity and temperature may affect our results and whether if the study was repeated at a different time of year, we would have got similar results.

This study does not assess the risk of hospital acquired infection related to different thermometry methods. This is the topic of previous research [24], and there have been notable cases of thermometer related hospital disease outbreaks [25,26]. We acknowledge that choice of routine thermometry method needs to include this consideration.

## 7 Conclusions

With limited diagnostic tools, resource-poor settings heavily rely upon patient history and clinical findings, including body temperature for disease recognition and risk stratification. Temperature measurement needs to be practical, accurate, acceptable and reproducible. This study concludes that NCITs lack the sensitivity and reproducibility to be a reliable method of fever screening in non-temperature-regulated tropical settings. Tympanic membrane thermometry may be a more suitable alternative but is currently not readily available in the region and also has limitations. Rectal thermometry is gold standard and has a good level of acceptability with staff and parents for use with children.

The limited sensitivity of all non-invasive thermometry methods needs to be acknowledged when designing and implementing triage protocols for high-risk infectious diseases such as VHF and COVID-19.

## Ethics statement

This study was reviewed and approved by the Sierra Leone National Ethics and Scientific Review Committee on 11 May 2021 (no document number issued); in addition, this study was reviewed and approved by the London School of Hygiene and Tropical Medicine (LSHTM)'s ethics committee (LSHTM MSc Ethics Ref: 25680). The LSHTM's Research Governance and Integrity Office kindly provided sponsorship. All participants or their legal guardians provided informed consent to participate in the study.

## Funding sources

Expenses at the MMRU were covered by MPG's research funds at the Center of Tropical Medicine and Travel Medicine, AUMC, Amsterdam.

## Data availability statement

Data have not been deposited in a publicly available repository, but are available upon request to the authors on the basis of an analytical plan to be agreed upon.

## CRediT authorship contribution statement

**Alexandra Turnbull:** Writing – review & editing, Writing – original draft, Formal analysis, Data curation, Conceptualization. **Harry Putnam:** Writing – review & editing, Writing – original draft, Formal analysis, Data curation. **Issa Sesay:** Writing – review & editing, Data curation. **Aminata Bangura:** Writing – review & editing, Data curation. **Emily Bailey:** Writing – review & editing, Data curation. **Jan Henk Dubbink:** Writing – review & editing, Writing – original draft, Data curation, Conceptualization. **Martin P. Grobusch:** Writing – review & editing, Writing – original draft, Validation, Supervision, Resources, Project administration, Methodology, Formal analysis, Data curation, Conceptualization.

## Declaration of competing interest

The authors declare that they have no known competing financial interests or personal relationships that could have appeared to influence the work reported in this paper.
