# Supplementary material for: So, what's best? Accuracy and acceptance of thermometers in triage and inpatients in a low-resource tropical setting – The MaTe study
Source: Heliyon. 2024 Feb 6;10(3):e25806. doi: 10.1016/j.heliyon.2024.e25806 (PMC10873737; doi:10.1016/j.heliyon.2024.e25806)
Supplement: Multimedia component 1 [file mmc1.pdf]

Supplementary Material

Supplementary Figure 1 – picture cards for consent process

Rectal Measurements:

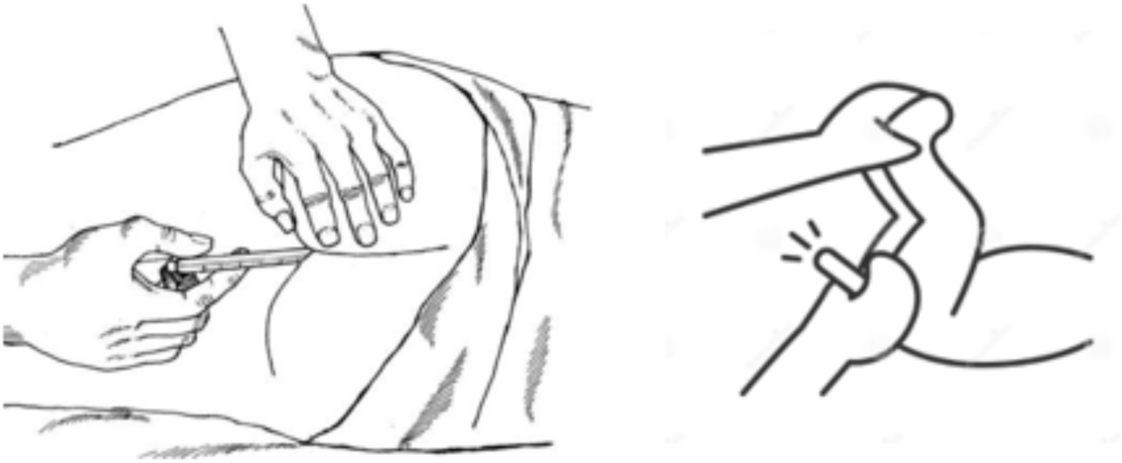

Ear (tympanic Membrane)

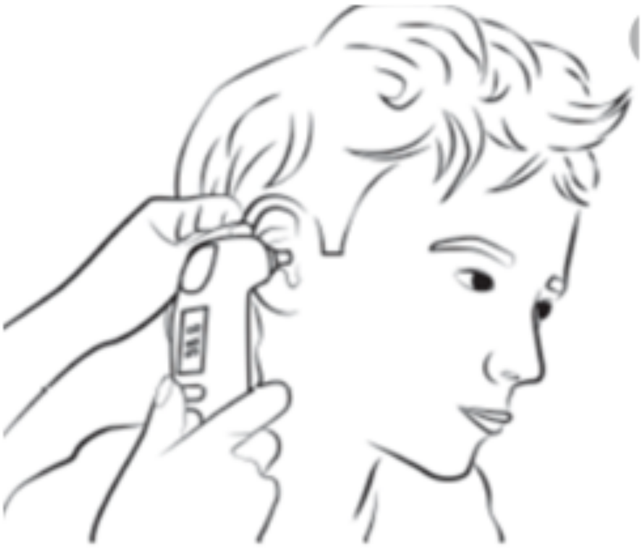

Axilla (Armpit) Temperature

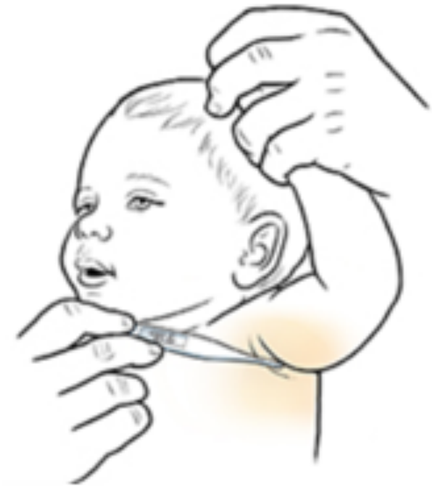

Infra-red Forehead Scanner

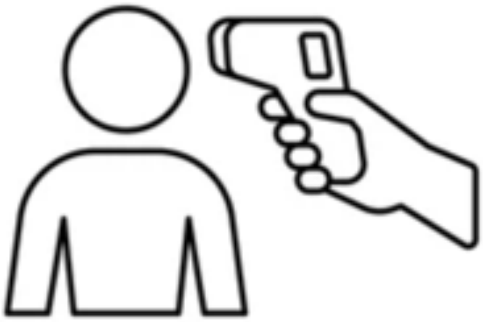

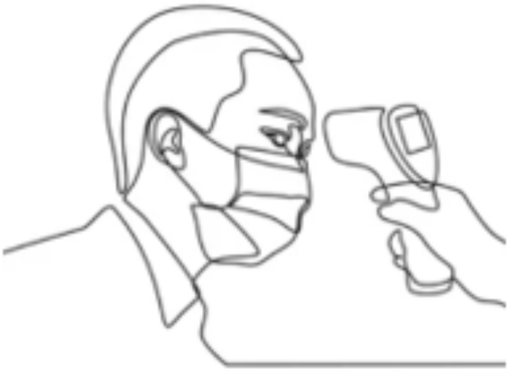

Supplementary Figure 2 – Full questionnaire and documentation for adult and children participants

1. What is the name of the data inserter?

2. What is the date today? (dd/mm/yy)

3. What is the participants study number?

4. Is the participant aged 1 year or above? (Please circle)

   Yes No

   If **no**: How many months old is the child?

   6 7 8 9 10 11

   If **yes**: How old is the participant?

   ……… years

5. What is the participants sex? (Please circle)

   Male Female Prefer not to say

   If **female** and **>14 years of age**: Does the participant know she is pregnant?

6. What was the temperature recorded at triage?

7. What is the rectal temperature?

8. What is the axillary temperature?

9. What is the ear (tympanic membrane) temperature? (Genius 3 - Dark Blue/White)

10. What is the ear (tympanic membrane) temperature? (Braun Pale Blue/White)

11. What is the forehead (infra-red) temperature? (Purple/White PC868)

12. What is the forehead (infra-red) temperature? (Blue/White MIGO)

13. Does the participant think, at current, they have a fever?

    Yes No

If participant < 15: Does the participants parent/legal guardian think, at current, their child has a fever?

Yes No

14. Please ask the participant the following: 'You have just had your temperature taken with 4 different methods. Please order your favourite'. (Rectal, axillary, ear, forehead)

Most favourite:
Second favourite
Third favourite:
Least favourite:

If participant < 15: Please ask the partipants parent/legal guardian the following: 'Your child has just had it's temperature taken 4 different ways, please order your favourite to least favourite way for your child' (Rectal, axillary, ear, forehead)

Most favourite:
Second favourite
Third favourite:
Least favourite:

15. Please ask the participant: 'Would you be happy for the nurse to take your temperature every day using the following methods?' (please circle)

Rectal :	Yes	No
Ear:	Yes	No
Axilla:	Yes	No
Forehead:	Yes	No

If participant < 15: Please ask the participants parent/legal guardian the following: 'Would you be happy for a nurse to take your childs temperature regularly using the following methods?'

Rectal :	Yes	No
Ear:	Yes	No
Axilla:	Yes	No
Forehead:	Yes	No

Supplementary Figure 3 – Full questionnaire and documentation for participating nursing staff

1. What is the name of the data inserter?

2. What is the date today? (dd/mm/yy)

3. What is the participants study number?

4. Which of the four methods discussed do you think yields the most reliable results?
   Rectal Axilla Ear/Tm Forehead Scanner

5. Which of the four discussed do you think yields the least reliable results?
   Circle one of: Rectal Axilla Ear/Tm Forehead Scanner

6. Please rank which method you think is easiest to explain to the patient?

Most easiest:
Second easiest:
Third easiest:
Least easiest:

7. Please rank which method you think is the most time effective?

Most time effective:
Second most time effective:
Third most time effective:
Least time effective:

8. Would you be happy to use the follow methods regularly on the ward for taking observations?

Rectal :	Yes	No
Axilla:	Yes	No
Ear:	Yes	No
Forehead:	Yes	No
